# Effect of Medical Interventions on Body Satisfaction in Gender-Dysphoric Adolescents

**DOI:** 10.1007/s10508-025-03329-6

**Published:** 2025-12-04

**Authors:** Camille Ammann, Lukasz Smigielski, Manuela Lutz, Tanja Schenker, Verena Riedo, Nicole Besse-Flütsch, Isabelle Häberling, Susanne Walitza, Dagmar Pauli

**Affiliations:** Department of Child and Adolescent Psychiatry and Psychotherapy, Psychiatric University Hospital Zurich, University of Zurich, Neumünsterallee 9, 8032 Zurich, Switzerland

**Keywords:** Body image, Body satisfaction, Gender dysphoria, Adolescents, Gender-affirming hormones, Gender-affirming surgery

## Abstract

Body dissatisfaction contributes to distress in gender dysphoria, but longitudinal evidence on factors alleviating this discomfort is limited. This naturalistic study examined the effect of gender-affirming hormone therapy (GAHT) alone and in combination with gender-affirming surgery (GAHT + GAS) on body satisfaction. Eighty-two adolescents (mean age, 15.77 ± 1.37) referred to a specialist clinic were examined at two time points and classified into three groups at follow-up (mean interval, 1.93 ± 0.72 years): (1) no medical interventions or puberty blocking (*n* = 15; counts of birth-assigned females and males: 11F/4M); (2) GAHT (*n* = 40; 28F/12M); and (3) GAHT + GAS (*n* = 27; 26F/1M). Group-level changes in Body Image Scale over time, nonmedical predictors, and associations between different facets of body dissatisfaction and life satisfaction were examined. Compared to the no-intervention/puberty blocking group, adolescents showed significantly reduced body dissatisfaction after gender-affirming interventions with no statistically significant difference between the GAHT and GAHT + GAS groups. Although there were no predictors of change in body dissatisfaction, social transitioning and satisfying family functioning at baseline were associated with lower body dissatisfaction at follow-up. Body dissatisfaction and life satisfaction were negatively correlated. These results suggest gender-affirming interventions can alleviate body dissatisfaction, at least in the short term. Moreover, social transitioning and family support were factors linked to body satisfaction. Future research should further investigate the benefit-risk profile of gender-affirming interventions in the psycho-functional and somatic domains, applying more robust study designs that minimize selection and response bias, with longer observation periods and larger sample sizes.

## Introduction

Body image is defined as the way one evaluates, feels about, and intervenes in one’s own embodiment, which extends to pure physical appearance (Cash, 1994). A beneficial role of holding positive attitudes toward one’s own body has been largely emphasized by a recent meta-analysis based on 240 published studies, which linked the construct of body appreciation with indices of well-being (positively) and psychopathology (inversely) (Linardon et al., 2022). It has also been shown that body satisfaction is associated with life satisfaction in men and women (Davis et al., 2020). In people with gender dysphoria (GD), defined in the fifth edition of the *Diagnostic and Statistical Manual of Mental Disorders* (DSM-5) as “the distress that may accompany the incongruence between one’s experienced or expressed gender and one's birth-assigned gender” (American Psychiatric Association, 2013), body dissatisfaction and uneasiness with physical characteristics are significantly more common compared to non-gender-dysphoric people (Algars et al., 2012; Bandini et al., 2013; van de Grift, 2016b; Witcomb et al., 2015). This also applies to children and adolescents (Becker et al., 2018; Fisher et al., 2017), although the DSM-5 diagnostic criteria of GD in children contain more behavioral than body dysphoria aspects compared to criteria defined for adolescents and adults. Steensma et al. (2013) found that body dissatisfaction was significantly higher in “persisters” (i.e., those who continue in the path of gender transition) than in “desisters” (i.e., those who revert to the birth-assigned gender when entering puberty), underlining the important role of body satisfaction in the course of GD (Steensma, 2013). People with GD also tend to experience high levels of psychopathology (Dhejne et al., 2016), self-injury (Davey et al., 2016; Marshall et al., 2016), and suicidality/suicidal ideation (Aitken et al., 2016; Bauer et al., 2015; de Graaf et al., 2022; Marshall et al., 2016; Ruuska et al., 2024; Wiepjes et al., 2020).

The thematic of satisfaction or dissatisfaction with one’s body is present in both healthy individuals and those experiencing mental health issues. A clinical condition with substantial research literature that has a strong focus on body satisfaction is eating disorders. Body dissatisfaction was found to be significantly associated with suicidality in anorexia nervosa and bulimia nervosa (Perkins & Brausch, 2019; Rufino et al., 2018). Several studies sought to compare the body dissatisfaction parameters in eating disorders with those of individuals with GD (Jones et al., 2016; Vocks et al., 2009). In the two aforementioned studies, people with GD exhibited less severe body dissatisfaction compared to those with eating disorders. However, in another study, gender dysphoric trans* men expressed similar levels of body dissatisfaction as non-gender-dysphoric cis males with eating disorders, possibly putting them at a greater risk of developing eating-related symptomologies (Witcomb et al., 2015). A recent study showed that body dissatisfaction was a significant predictor of psychological functioning in adolescents with GD, both for internalizing and externalizing problems (Verveen et al., 2023). Overall, these findings suggest that body dissatisfaction can influence psychosocial functioning in gender dysphoric persons, but also in those with eating disorders, highlighting its potential relevance as a topic of clinical focus.

While most treatments for eating disorders focus initially on normalizing eating patterns, body dissatisfaction can be addressed by promoting positive emotion regulation, healthy weight control beliefs, and psychoeducation (Laporta-Herrero et al., 2018). Various medical interventions have been proposed to minimize the feeling of incongruence between one’s experienced or expressed and one’s birth-assigned gender in GD. Several studies indicate that body dissatisfaction decreased in GD after gender-affirming hormone therapy (Becker et al., 2018; Kuper et al., 2020; van de Grift et al., 2017) and after gender-affirming surgery (Kraemer et al., 2008; Robinson et al., 2021; Smith et al., 2005). In a study by de Vries et al. (2014), puberty-suppression alone had no impact on the extent of body dissatisfaction in children and adolescents. In contrast, gender-affirming hormone therapy and surgery significantly reduced the extent of both GD and body dissatisfaction. However, this study did not examine which of the interventions or whether the combination of treatments alleviated body dissatisfaction (de Vries et al., 2014). Body changes caused by hormone therapy were addressed in a study by Klaver et al. (2018), who found significant alterations in the waist-hip ratio, total body fat, and total lean body mass in both trans* males and trans* females, leading to a body composition more similar to their affirmed gender (Klaver et al., 2018). In another study, van de Grift et al. (2017) demonstrated that gender-affirming hormone therapy not only affects dissatisfaction with hormone-influenced body regions, but also with hormone-unresponsive regions such as the hand, calf, or nose. This implies that the improvement in body satisfaction through medical interventions may not be limited to the direct bodily effects of hormones or surgery but also seems to produce an overall more positive evaluation of oneself and one’s body. In summary, the published literature has shown a consistent association between GD and body dissatisfaction, with some studies focusing on the improvement of body satisfaction over the course of medical interventions. However, research examining the isolated effect of different medical interventions is sparse. Additionally, study populations were small in previous investigations, limiting their conclusions.

Next to medical interventions, social factors have also been proposed as factors potentially alleviating psychological distress in GD. For example, Durwood et al. (2017) found that socially transitioned youth with GD reported similar levels of depression and anxiety as controls without GD. This may indicate a positive effect of having socially transitioned (i.e., presenting oneself to and interacting with society in alignment with one’s felt gender identity) on psychopathology. However, the opposite causality is also conceivable: individuals free of depression and anxiety may express themselves as they see fit, compared to those constrained by their psychopathology (Durwood et al., 2017). In other studies, having socially transitioned did not predict improved psychological functioning, while high family functioning and peer relations were strong predictors of psychological well-being (Sievert et al., 2021; Wong et al., 2019). These conclusions were reached previously by Bauer et al. (2015), who found that social support, low levels of societal transphobia, and parental support of gender identity reduced suicide risk in trans* persons. The importance of peer support in the context of body satisfaction has also been demonstrated in eating disorder research, indicating that alienation from peers was associated with body dissatisfaction (Laporta-Herrero et al., 2021).

Although various previous studies have examined the association between gender-affirming medical interventions and body satisfaction, few have used a longitudinal design, and even fewer have focused on comparing outcomes in individuals with GD who received only hormone interventions or both hormone and surgical interventions. The main objective of the current study was to examine the effects of medical interventions on body satisfaction development in gender dysphoric adolescents over time in a naturalistic setting. Additionally, we aimed to identify non-medical predictors of body dissatisfaction at follow-up and its change. Based on the previous literature and our own clinical experience, our set of non-medical predictors included peer support, family functioning, social transition, birth-assigned gender, and age, as well as body mass index (BMI). The inclusion of BMI was motivated by its suggested association with body satisfaction in adolescents (Loth et al., 2015). Moreover, we examined the associations between more specific body dissatisfaction facets (i.e., primary, secondary, and neutral sex characteristics) and life satisfaction. We hypothesized that participants who received gender-affirming hormone therapy or surgery would show a significant improvement in body satisfaction over time, compared with persons who received no medical intervention or only puberty blockers. Moreover, we expected that additional surgery would result in a significantly greater reduction in body dissatisfaction than gender-affirming hormone treatment alone. Furthermore, we anticipated that non-medical psycho-social factors, such as family functioning, peer relationships, and social transition status, would be significant predictors of change in body satisfaction over time. In our correlation analysis, we expected to see a positive correlation between body dissatisfaction and the extent of GD and hypothesized that dissatisfaction with primary sexual characteristics such as genitals would show the strongest correlations with overall body dissatisfaction. Given the previously identified association between positive constructs, such as well-being and life satisfaction, we hypothesized that life satisfaction would be negatively correlated with body dissatisfaction. As the number of referrals to GD clinics is increasing worldwide (Handler et al., 2019), elucidating these issues is of high relevance for transgender youth, their parents, and clinicians.

## Method

### Participants

**Fig. 1:**
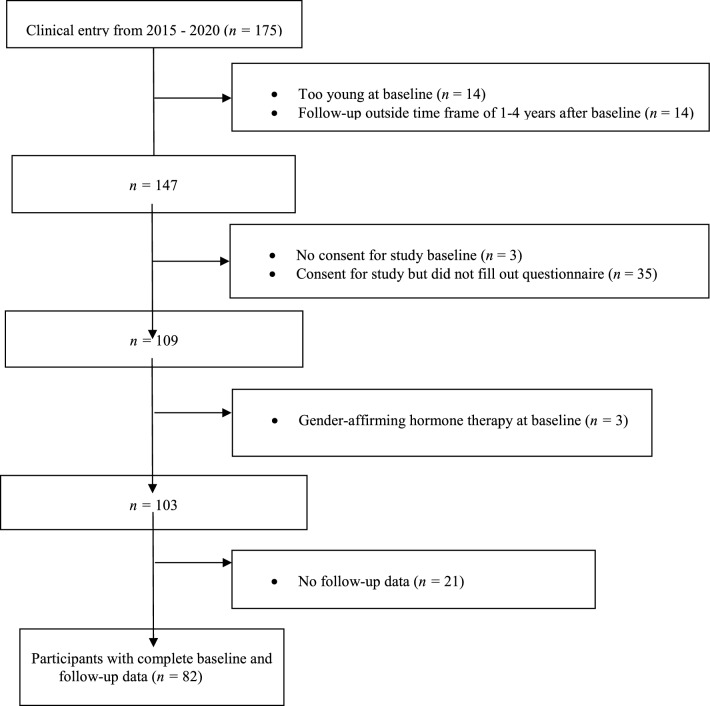
Participant flowchart with reasons for exclusion

This study was part of the longitudinal naturalistic cohort research project “Variations in the development of gender identity in children and adolescents” launched by the Gender Clinic of the Department of Child and Adolescent Psychiatry and Psychotherapy of the Psychiatric University Hospital Zurich. This specialist service was established in 2009, and data collection started in 2015. Figure 1 depicts the participant flowchart with reasons for exclusion. Out of 175 clinical entries, 28 adolescents did not meet inclusion criteria because of age (< 12 years old) or a follow-up date outside the set time frame of 1–4 years. Although cognitive impairment leading to an inability to complete the questionnaires was an exclusion criterion, no individual was excluded from the study on this basis. Of the 147 adolescents who qualified for our study, 109 filled out the baseline questionnaire (participation rate 74.2%). However, six adolescents had to be excluded because of already initiated gender-affirming hormone treatment or desisting (i.e., identifying with one’s birth-assigned gender again). Of the remaining 103 adolescents, 21 did not fill out the follow-up questionnaires, leading to a participation rate of 79.6% at follow-up and a final number of complete datasets of 82 (total participation rate: 58.2%).

### Procedure

In our analyses of this naturalistically collected data, we included two timepoints for each participant, whereby the follow-up time point (T1) occurred in the time frame of 1–4 years, independent of possibly initiated medical interventions. This led to significantly differing time intervals between baseline (T0), the start of possible medical interventions, and follow-up (T1), which is why time was modeled in the longitudinal analysis. Participants were required to be treatment-naïve (i.e., not receiving any gender-affirming healthcare) at timepoint T0. At T1, participants were classified into one of three groups, dependent on the medical treatment they had received between T0 and T1. This resulted in three study groups: (1) no medical intervention or puberty blocking; (2) gender-affirming hormone treatment (GAHT); and (3) gender-affirming hormones and surgery (GAHT + GAS). On average, T1 data were collected 14.30 months (± 8.62) after the start of gender-affirming hormone treatment and 11.89 months (± 8.37) after gender-affirming surgery. (This datum was available for 90.04% and 70.37% of participants, respectively.) Instruments measuring the constructs of interest were administered at either one or both timepoints, as specified below. The decision regarding whether and which medical intervention was chosen for each person was made through an individual shared decision-making process. This process involved the adolescents, their parents, and specialized therapists, who evaluated the advantages and disadvantages of each potential intervention for the patient. Medical or surgical interventions were indicated for individuals with persistent, stable GD over several years, a substantial level of suffering, and on the basis of informed consent by the adolescent and guardian. At enrollment, 78 participants met the DSM-5 diagnostic criteria for GD, as assessed by trained clinicians. In four adolescents, the diagnostic criteria were not fully met at enrollment but were fulfilled shortly afterward, well before they began gender-affirming hormones or surgery. (Three of these participants did not receive medical interventions, while one received gender-affirming hormones and surgery.) All participants were thoroughly assessed over several months in supportive and diagnostic psychological sessions. Throughout the study, 68.3% (*n* = 56) of participants continued regular supportive psychotherapy: 12.2% (*n* = 10) attended once a week, 32.9% (*n* = 27) attended once every 2–4 weeks, and 23.2% (*n* = 19) attended less than once every 4 weeks. Additionally, participants could access laser therapy for facial or body hair removal and attend speech therapy if desired.

### Measures

#### Body Image Scale

Body (dis-)satisfaction was measured using the Body Image Scale (BIS), created specifically for transgender individuals (Lindgren & Pauly, 1975) and broadly used in prior studies on GD (Gümüşsoy et al., 2022; Huisman et al., 2022; Verveen et al., 2023). Its two versions (one for transgender males, one for transgender females) list 30 body features to be rated by participants on a response scale ranging from 1 to 5, where 1 indicates “most satisfied” and 5 indicates “most dissatisfied.” According to the original authors Lindgren and Pauli, all BIS items are subdivided into three subscales: primary sex characteristics (genitals, breasts, facial hair; voice and chest in trans* males; body hair in trans* females), secondary sex characteristics (e.g., hips, thighs, arms, waist, muscles, buttocks, etc.), and neutral sex characteristics (e.g., nose, eyebrows, chin, calves, hands, etc.). Scoring was performed by calculating the mean of all items for the sum score and of the items loading the subscales, whereby a higher score represents greater body dissatisfaction. This measure was administered at T0 and T1.

#### Utrecht Gender Dysphoria Scale

The UGDS is a validated instrument used to measure the extent of GD (Cohen-Kettenis & van Goozen, 1997). The two versions of the questionnaire, for male-to-female and female-to-male transgender individuals, each contain 12 items, which are rated on a 5-point Likert scale, where 1 indicates “complete disagreement” and 5 indicates “complete agreement.” The total score is calculated by summing all item scores, resulting in a minimum of 12 and a maximum of 60 points, whereby higher scores indicate stronger GD. A cutoff point of 40 is recommended as a diagnostic threshold for GD (Steensma, 2013). This measure was administered at T0.

#### Other Instruments

Family functioning was assessed with the General Functioning 12-item subscale of the McMaster Family Assessment Device (FAD) (Epstein et al., 1983). The items are rated on a 4-point Likert scale (where 1 indicates “best functioning” and 4 indicates “worst functioning”), from which a mean score is calculated (Boterhoven de Haan et al., 2015). Furthermore, the German version of the Youth Self-Report (YSR) (Achenbach, 1991; Döpfner et al., 1994) was used to derive the Poor Peer Relations (PPR) index by adding the responses to three YSR Items, namely Items 25 (“Doesn’t get along with other kids”), 38 (“Gets teased a lot”), and 48 (“Is not liked by other kids”), as suggested previously (Zucker et al., 1997) and used in other research in the field (de Graaf et al., 2018). The PPR total score ranges from 0 to 6, where higher scores indicate worse peer relations. Social transition status was assessed with the question “Are you living as your affirmed gender in all parts of your life?” with “yes” or “no” as dichotomous answers. Life satisfaction was measured with the 5-item Satisfaction with Life Scale (SWLS) (Diener et al., 1985), where each item is rated from 1 to 7, according to the extent of agreement with the statement. The SWLS total score ranges from 5 to 35 and is calculated by summing up all items, whereby higher values indicate higher satisfaction with life. The PPR score was used at T0 and T1, FAD was used at T0 only, and SWLS was used at T1 only. BMI was measured using the formula: weight (kg) divided by height squared (m^2^). Participants’ weight was recorded with a calibrated scale, and height was measured with a stadiometer.

### Statistical Analysis

All statistical analyses were conducted in R version 4.1.1 (R Core Team, 2021). To evaluate associations among groups, Pearson chi-square test/Fisher’s exact test was used for categorical data and ANOVA/Kruskal–Wallis tests were applied to continuous data, according to conventional statistical assumptions. The research questions were examined using three main statistical analyses. First, to test for possible between-group differences in the outcome variables BIS across two assessments, a linear mixed-effects modeling framework with a random intercept structure was applied. We considered this approach superior to the standard repeated-measures ANOVA, in particular because of the varying individual time intervals between the pairs of measurements. Mixed-effect models also account for between-subject variability and correlations within the data, while also adequately handling unbalanced and missing data (Baayen et al., 2008). We fitted a robust linear mixed-effects model (RLMM) by means of the “robustlmm” package (Koller, 2016). Group (i.e., no GAT/puberty blocking, GAHT, GAHT + GAS), time (in years), birth-assigned gender, age, and the group-by-time interaction were modeled as fixed effects, and the intercept for the participants was modeled as a random effect. Specifically, we tested the hypothesis that the participants in the GAHT and GAS groups showed decreases in body dissatisfaction compared to the no-intervention/puberty blocking group from pre- to post-test. Satterthwaite-approximated degrees of freedom were used to generate *p*-values. No imputation of missing data was applied.

Our second aim was to test whether the non-medical measures (especially socially determined measures) collected at baseline significantly predicted the BIS score at the follow-up assessment, as well as its change score (T0–T1). The following predictors were included in the model: birth-assigned gender, age, BMI, FAD, PPR, and social transition. These analyses were performed using the robust linear regression function integrated within the “MASS” package in R. The functions from the “repmod” and “sfsmisc” packages were applied to derive *p*-values and robust *F*-test statistics, respectively. The tables of results for the above analyses were generated using the “sjPlot” package in R (Lüdecke, 2021).

Third, correlational analyses were conducted to examine the associations among the key study variables, including subscale scores. Specifically, Spearman’s rank-order correlations were calculated separately for T0 and T1 data between the BIS mean score, BIS subscales for primary, secondary, and neutral sex characteristics, UGDS (T0) and SWLS (T1), with Bonferroni corrections applied to account for multiple testing (20 tests performed, corresponding to a threshold of *p* = 0.0025).

## Results

### Sample Characteristics

No statistically significant differences were observed in basic demographics between non-participants and participants, including age (mean 15.83 ± 1.53 for non-participants; mean 15.71 ± 1.40 for participants; *t*(156) = 0.53, *p* = 0.600), birth-assigned sex (*χ*^*2*^ = 1.03, *p* = 0.311), and the presence of comorbidities (*χ*^2^ = 3.48, *p* = 0.062). Complete data for these variables were available for 76 non-participants. Table 1 summarizes participant characteristics. The mean age for the whole sample (*n* = 82) at baseline was 15.77 years (± 1.37, age range 12–18 years). The time interval between baseline and follow-up assessments varied from 0.93 to 3.71 years with a mean of 1.93 years (± 0.72) and significantly differed among the three analyzed groups; this was owing to an overall longer time interval between T0 and T1 in the surgery group. Nearly 80% of participants were birth-assigned females. At baseline T0, none of the participants had undergone gender-affirming hormone treatment or surgery. Eight adolescents had received puberty-suppressing hormones (i.e., gonadotropin-releasing hormone [GnRH] analogs) at baseline. The surgery group contained significantly more birth-assigned females than the other two groups (26 out of 27 gender-affirming surgeries were mastectomies; the remaining was a genital reassignment surgery in a birth-assigned male). All participants in our study, except for two, had UGDS scores over the suggested diagnostic cutoff of 40 points at baseline. Said two participants had scores of 35 and 38 points, respectively, and nevertheless checked all criteria for the diagnosis of GD according to DSM-5. While there was no statistically significant difference in the presence/absence of psychiatric comorbidities across the groups, the BIS score at baseline was significantly higher in patients with comorbidities (*n* = 30; mean 3.60 ± 0.40) compared to those without comorbidities (*n* = 52; mean 3.36 ± 0.50; *t*(80) = 2.19, *p* = 0.032).

**Table 1:** Summary of participant characteristics

	NoGAT/puberty blocking (*N* = 15)	GAHT (*N* = 40)	GAHT + GAS (*N* = 27)	Total (*N* = 82)	*p*
T0 measures					
Birth-assigned sex at birth *n* (%)					0.028^a,1^
N-Miss	0	0	0	0	
Male	4 (26.7%)	12 (30.0%)	1 (3.7%)	17 (20.7%)	
Female	11 (73.3%)	28 (70.0%)	26 (96.3%)	65 (79.3%)	
Age (years)					0.240^b^
N-Miss	0	0	0	0	
Mean (SD)	15.28 (1.62)	15.77 (1.28)	16.03 (1.33)	15.77 (1.37)	
BMI					0.699^c^
N-Miss	5	4	1	10	
Mean (SD)	21.37 (3.58)	22.76 (4.96)	21.26 (2.35)	22.03 (4.03)	
BIS mean score					0.001^b,2^
N-Miss	0	0	0	0	
Mean (SD)	3.08 (0.56)	3.47 (0.41)	3.63 (0.44)	3.45 (0.48)	
UGDS total score					0.048^c,3^
N-Miss	3	7	2	12	
Mean (SD)	53.41 (5.04)	55.00 (5.92)	56.76 (3.79)	55.36 (5.17)	
FAD score					0.490^c^
N-Miss	0	0	0	0	
Mean (SD)	2.20 (0.80)	2.09 (0.70)	1.90 (0.52)	2.05 (0.67)	
PPR score					0.378^c^
N-Miss	0	1	2	3	
Mean (SD)	1.47 (1.73)	1.28 (1.19)	0.88 (1.05)	1.19 (1.27)	
Soc. transition *n* (%)					0.164^a^
N-Miss	0	0	1	1	
Yes	2 (13.3%)	13 (32.5%)	4 (15.4%)	19 (23.5%)	
No	13 (86.6%)	27 (67.5%)	22 (84.6%)	62 (76.5%)	
YSR					
N-Miss	0	1	1	2	
Total score					
Mean (SD)	58.13 (26.23)	59.82 (20.38)	59.00 (18.21)	59.24 (20.66)	0.963^b^
Internalizing					
Mean (SD)	20.80 (10.60)	21.41 (9.87)	22.96 (10.02)	21.80 (9.96)	0.759^b^
Externalizing					
Mean (SD)	12.98 (5.82)	13.62 (6.87)	12.08 (5.11)	12.99 (6.12)	0.616^b^
Comorbidity					
No/yes	12/3	26/14	14/13	52/30	0.185^d^
T1 measures					
Age (years)					0.007^b,4^
N-Miss	0	0	0	0	
Mean (SD)	16.99 (1.95)	17.40 (1.39)	18.43 (1.54)	17.66 (1.63)	
BIS mean score					0.176^b^
N-Miss	0	0	0	0	
Mean (SD)	3.29 (0.54)	3.02 (0.54)	2.96 (0.58)	3.05 (0.56)	
SWLS					0.638^b^
N-Miss	0	3	1	4	
Mean (SD)	20.20 (5.31)	20.41 (6.64)	21.81 (6.71)	20.83 (6.39)	
Time difference T1–T0 (years)					< 0.001^c,5^
N-Miss	0	0	0	0	
Mean (SD)	1.71 (0.75)	1.70 (0.65)	2.40 (0.56)	1.93 (0.72)	

BIS, Body Image Scale; BMI, Body Mass Index; FAD, McMaster Family Assessment Device; GAHT, gender-affirming hormone therapy; GAHT + GAS, gender-affirming hormones and surgery; N-Miss, number of missing data sets per variable; *p*, level of significance with values in bold indicating *p* < 0.05; PPR, poor peer relations scale; Soc. Trans., socially transitioned; SD, standard deviation; SWLS, Satisfaction with Life Scale; UGDS, Utrecht Gender Dysphoria Scale; YSR, Youth Self-Report. The average SWLS values fall within the “slightly satisfied” range (21–25) according to the established norms. ^a^Fisher's exact test; ^b^ANOVA; ^c^Kruskal–Wallis test; ^d^Pearson chi-square test. Significant (*p* < 0.05) post hoc tests: ^1^GAHT vs. GAHT + GAS (Holm’s correction); ^2^No GAT/PB vs. GAH, No GAT/PB vs. GAHT + GAS (Tukey’s correction); ^3^No GAT/PB vs. GAHT + GAS (Holm’s correction); ^4^No GAT/PB vs. GAHT + GAS, GAHT vs. GAHT + GAS (Tukey’s correction); ^5^No GAT/PB vs. GAHT + GAS, GAHT vs. GAHT + GAS (Holm’s correction)

### Group-level Longitudinal Analysis

**Fig. 2:**
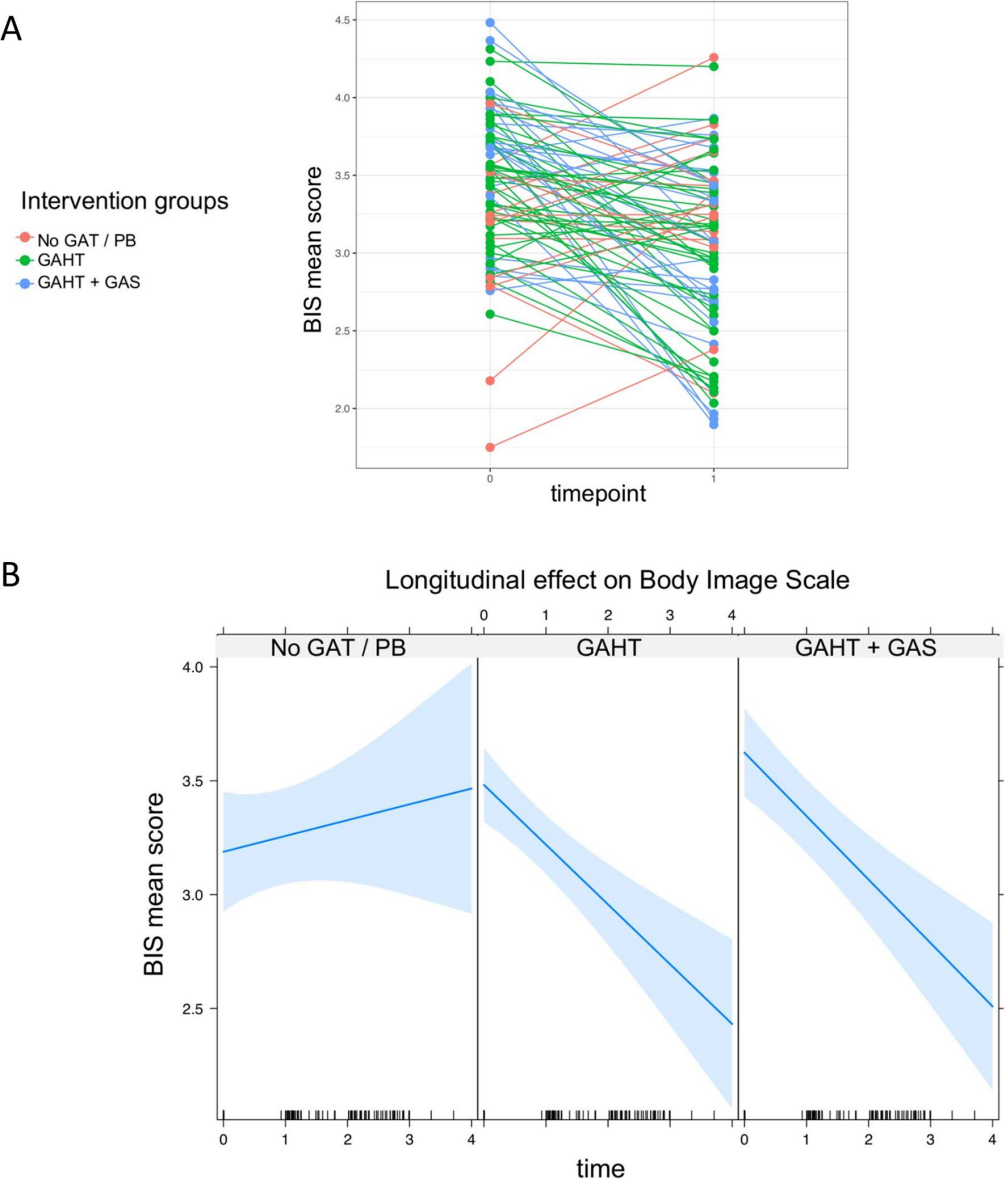
Plots of longitudinal effects on the Body Image Scale (BIS). A: Individual trajectories for BIS. Groups (as present at the T1 assessment) are marked in the same color. B: Group-by-time effect plot. Blue lines are the predicted slopes for BIS with corresponding 95% confidence intervals. Abbreviations: GAHT, gender-affirming hormone therapy; GAS, gender-affirming surgery; No GAT, no gender-affirming therapy; PB, puberty blocking

We hypothesized a significant reduction in BIS scores over time among participants receiving gender-affirming care, with further improvement expected following surgery. Among the key findings in this analysis (Fig. 2, Table 2), there were significant group-by-time interactions for the GAHT group (*β* = − 0.33, *p* < 0.001) and the GAHT + GAS group (*β* = − 0.35, *p* < 0.001). Compared to the no-intervention/puberty blocking group, the GAHT group and the GAHT + GAS group had 0.33- and 0.35-unit decreases per year in the outcome variable BIS (controlling for sex and age), respectively. In other words, there was a significantly greater improvement in body satisfaction in the GAHT and the GAHT + GAS groups. To test for possible differences between the GAHT and the GAHT + GAS groups, we repeated these two analyses by referring to the GAHT group as the reference level and found no statistically significant effects between the two intervention groups. In addition, we separately tested the effects of BIS subscales (primary, secondary, neutral sex characteristics) and again found significant interactions when the no-intervention/puberty blocking group was used as a reference, as well as no significant difference between the GAHT group and the GAHT + GAS group throughout all three BIS subscales. Given that 96.3% of participants in the GAHT + GAS groups were birth-assigned females receiving mastectomies, we conducted the same analyses only for this population. No statistically significant difference between the GAHT and GAHT + GAS groups could be found. The model had a marginal coefficient of determination (*R*^2^) of 0.230 and a conditional *R*^2^ of 0.484, meaning that the fixed effects explained 23% of the variance, while the combination of fixed and random effects (i.e., the full model) explained 48.4% of the variance. The sample size in this study was determined by the available data, and no a priori power calculation was conducted. However, we performed a post hoc power calculation for the main result in the pre–post-group analysis. Specifically, using the simr package for R (Green & MacLeod, 2016) and 1000 iterations, we demonstrated a simulated statistical power of 71.3% [95% CI 68.4–74.1%] for the interaction term.

**Table 2:** Results of the group analysis for the Body Image Scale

*Predictors*	*Est.*	Body image *CI*	*p*
(Intercept)	2.81	1.76 to 3.86	< 0.001
Group [GAHT]	0.29	0.01 to 0.58	0.046
Group [GAHT + GAS]	0.44	0.12 to 0.75	0.007
Time	0.07	-0.10 to 0.23	0.412
Age	0.02	-0.05 to 0.08	0.597
Birth-assigned gender [female]	0.11	-0.12 to 0.33	0.359
Group [GAHT] * time	-0.33	-0.51 to -0.15	< 0.001
Group [GAHT + GAS] * time	-0.35	-0.52 to -0.17	< 0.001
Random effects			
* σ*^2^	0.15		
* τ*_00 ID_	0.08		
ICC	0.33		
*N*_ID_	82		
Observations	164		
Marginal *R*^2^/Conditional *R*^2^	0.230/0.484		

CI, confidence interval; Est., beta estimate; GAHT, gender-affirming hormone therapy; GAHT + GAS, gender-affirming surgery; ICC, intraclass correlation coefficient; N _ID_, number of participants; p, level of significance with values in bold indicating p < 0.05; R2, coefficient of determination; *σ*^2^, within-person variance; τ00 ID, between-person variance. The reference level in this analysis is the group without gender-affirming therapy or puberty blocking (No GAT/PB)

### Predictor Analysis

We hypothesized that family functioning, peer support, and social transition status would be significant predictors of BIS and its change over time in the whole study sample. The variance inflation factor (VIF) in the predictor analyses was between 1.03 and 1.19, indicative of no multicollinearity issues among predictors. The overall regression model involving the BIS (T1) as the outcome variable was statistically significant (robust *F* = 3.609,* p* = 0.004). The two significant predictors in this model were social transition status (*β* = − 0.57, *p* < 0.001) and family functioning (FAD, *β* = − 0.21, *p* = 0.044). Those participants who had socially transitioned and had higher levels of family functioning at T0 tended to be less dissatisfied with their bodies at T1. No statistically significant predictor was identified for the BIS change scores; however, there was a trend toward significance for social transitioning among the predictors (Table 3).

**Table 3:** Non-medical predictor analysis for the Body Image Scale in the whole study sample

Predictors	Body Image Scale (T1)			Body Image Scale (change T1–T0)		
	*Est.*	*CI*	*p*	*Est.*	*CI*	*p*
(Intercept)	3.23	1.49 to 4.96	< 0.001	− 1.23	− 3.35 to 0.89	0.244
Birth-assigned gender [f]	0.05	− 0.27 to 0.38	0.748	0.10	− 0.30 to 0.50	0.631
Age (T0, years)	− 0.07	− 0.17 to 0.04	0.214	0.11	− 0.02 to 0.23	0.103
BMI (T0)	0.03	− 0.01 to 0.06	0.108	0.01	− 0.03 to 0.05	0.592
FAD (T0)	0.21	0.00 to 0.42	0.044	− 0.18	− 0.43 to 0.07	0.157
PPR (T0)	− 0.02	− 0.13 to 0.09	0.723	− 0.04	− 0.17 to 0.09	0.562
Soc. trans [yes]	− 0.57	− 0.88 to 0.27	< 0.001	0.36	− 0.01 to 0.73	0.054
Observations	68	68				

BMI, Body Mass Index; *CI*, confidence interval; *Est.*, beta estimate; f, female; FAD, McMaster Family Assessment Device; *p*, level of significance with values in bold indicating *p* < 0.05; PPR, Poor Peer Relations scale from the Youth Self-Report

### Correlation Analysis

**Fig. 3:**
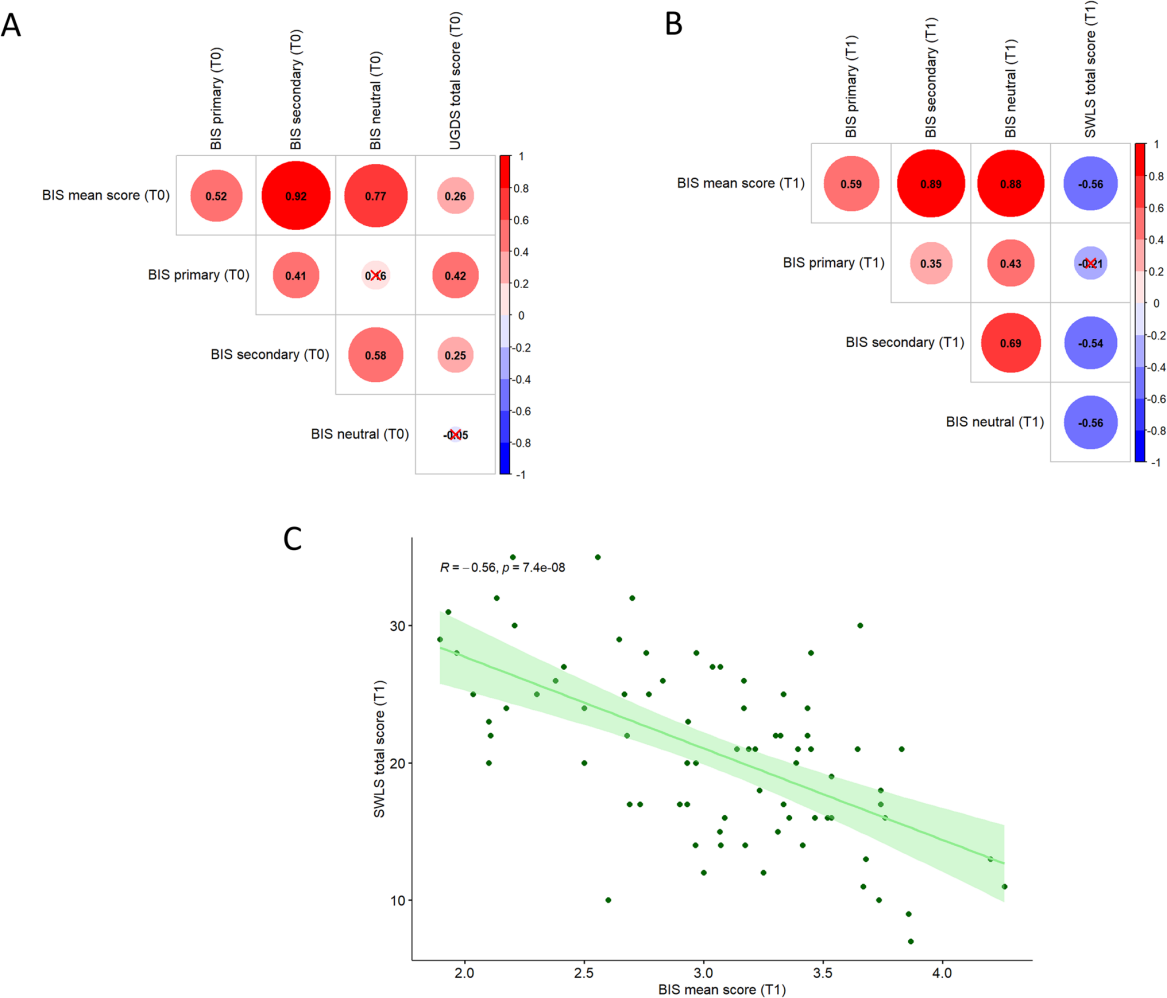
Correlations between the Body Image Scale (BIS), its subscales, Utrecht Gender Dysphoria Scale (UGDS), and Satisfaction with Life Scale (SWLS). Spearman correlation matrix with significant positive and negative correlations after Bonferroni correction displayed as red and blue circles, respectively, for available T0 (**A**) and T1 (**B**) data. The uncrossed effects are statistically significant (after correction). **C** Spearman correlation plot for BIS and SWLS (T1)

The Bonferroni-corrected Spearman correlation matrix (Fig. 3A–B) revealed positive associations within the BIS mean score and subscale scores for both timepoints, except for the pair of the BIS primary and BIS neutral dimensions at T0. The strongest correlation was found between the BIS mean score and BIS secondary characteristics (*ρ* = 0.92, *p* < 0.001). There were also significant correlations involving the BIS and UGDS at T0, except for the BIS neutral sex characteristics. Importantly, we also found significant negative associations between life satisfaction (SWLS) and BIS mean scores (Fig. 3B, *ρ* = − 0.56, *p* < 0.001). Notably, life satisfaction was negatively correlated with the secondary and neutral BIS subscales (secondary, *ρ* = − 0.54, *p* < 0.001; neutral, *ρ* = − 0.56, *p* < 0.001), but not with the BIS primary subscale (*ρ* = − 0.21, *p*_*uncorrected*_ = 0.06).

## Discussion

As the main result, the present study found that, in adolescents with GD, gender-affirming hormone therapy and hormone therapy combined with surgery were linked to a statistically significant reduction in body dissatisfaction over time, compared to no medical intervention. Overall, this effect aligns with the findings from previous research on the effects of gender-affirming interventions in adolescents with GD (de Vries et al., 2014; Kuper et al., 2020). The effects of gender-affirming hormones consist of biological changes in body shape, hair, and voice. Surgery modifies the birth-assigned-gender-typical body parts and can construct body parts typical of the affirmed gender. Unexpectedly, no significant difference in the reduction of body dissatisfaction was found between the hormones-only group and the hormones and surgery group in our work. This was the case for all three BIS subscales and was consistent with the subgroup analysis with birth-assigned females only, as observed in our additional analyses. Few studies have investigated the additional effect of gender-affirming surgery on the reduction of body dissatisfaction in GD, compared to the effect of hormone therapy alone. For example, a study with 201 participants by van de Grift et al. (2017) found no significant difference in the extent of overall body dissatisfaction at follow-up assessments between the hormones-only group and the hormones and surgery group. Only the subgroup analysis of genital satisfaction, which was assessed in one of six newly created BIS subscales, was higher in the hormones and surgery group. These groups, however, were of very different sizes, with 136 participants in the hormones and surgery group and only 36 participants in the hormones-only group (van de Grift et al., 2017). On the other hand, a recent study examining 70 individuals showed that mastectomy in trans* males or non-binary persons who had already undergone gender-affirming hormone therapy led to a reduction in chest dysphoria and overall body dissatisfaction (Ascha et al., 2022). A potential explanation for the lack of a difference between these two groups in our study could be that the participants were still recovering from surgeries and the follow-up assessment occurred too soon after gender-affirming surgery (mean time difference 11.89 months, ± 8.37), such that possible surgical wounds or pain may have negatively affected body satisfaction. It should also be noted that the reasons why participants in the untreated group did not receive medical treatment are diverse. Some had not sought treatment because they were uncertain; in some cases, the parents did not consent; for others, the care providers had not established an indication for treatment, e.g., because of a lack of stability of GD.

In the predictor analysis, no statistically significant effects were found for the change score of the BIS. There was a trend effect for social transition (*p* = 0.054). However, both social transition status and family functioning level at baseline were significant predictors of body satisfaction at follow-up assessments. This aligns with the view that gender affirmation and good relationships with relatives may support a positive body image and make gender-affirming interventions more accessible in the first place. Social transitioning has been described as a positive factor for psychological well-being in GD in the previous research (Durwood et al., 2017). However, it is important to note that the social transition was evaluated in our study with a simple yes-or-no question, which is a subjective measure of whether someone is living in their desired role and may vary depending on the participant's mood or life situation. Family functioning was recorded using the FAD, which measures aspects such as support in difficult situations, mutual acceptance, and trust. Generally, high family functioning is associated with better mental health, manifested as reduced levels of stress, depression, or anxiety in adolescents without GD (Lema-Gómez et al., 2021). Poor family functioning has also been shown to be a predictor of behavioral and emotional difficulties in adolescents with GD (Levitan et al., 2019). While our results point to predictors of body satisfaction at follow-up and not of the change, they may still be of interest for clinicians at the admission timepoint, and they also overall harmonize with the existing literature both in GD and mixed adolescent populations.

Regarding the correlation analysis, a few remarks can be made. At baseline, the extent of GD measured with the UGDS was correlated with the BIS facets except for the neutral BIS subscale, suggesting that the BIS can capture some level of GD. The BIS mean score, the primary measure assessed in our analyses, was highly correlated with secondary and neutral body characteristics and less correlated with primary body characteristics at baseline and at follow-up. This suggests a stronger association with more noticeable and general body aspects, such as stature or muscles, compared to primary sex characteristics that are altered by gender-affirming surgery. A similar observation was made for adults with gender dysphoria by van de Grift et al. in 2016, whereby socially influential body characteristics, such as voice, muscularity, and posture, had more influence on overall body satisfaction than genitals (van de Grift, 2016a). Notably, our study also found an association between the satisfaction with one’s body and life satisfaction. Such a relationship has been described in previous studies in the general population (Swami & Todd, 2022). Here again, a facet-specific pattern was apparent; life satisfaction was negatively correlated with dissatisfaction with both secondary and neutral sex characteristics (to similar degrees), but not primary sex characteristics, as measured by the BIS. This underlines the relevance of less- or non-hormone-responsive body parts as drivers of life satisfaction in gender dysphoric adolescents. It should be noted that the correlation does not prove causality; it is also possible that higher levels of life satisfaction lead to greater body satisfaction, rather than the other way around.

In light of the results from these studies, one might hypothesize that gender-affirming hormones and/or gender-affirming hormones and surgery may have the same positive effect on psychosocial functioning in children or adolescents with GD. However, recent systematic reviews (Ludvigsson et al., 2023; Taylor et al., 2024) have shown that there is a lack of high-quality studies on the psychological and mental health outcomes of adolescents with GD treated with puberty blockers or gender-affirming hormones. Accordingly, no final conclusions can be drawn at this stage, and more studies are needed in the future. In addition, possible adverse events are and should continue to be the subject of ongoing discussion and investigation. In the case of GnRH analogs, available evidence on their effect on bone mineralization is discordant and incomplete (Saggese et al., 1993). Several studies show unchanged absolute bone density, accompanied by a decrease in bone turnover parameters and lowering of bone mineral density *z*-scores (Navabi et al., 2021; Vlot et al., 2017). These findings suggest compromised bone development in youth under puberty suppression compared to peers of the same birth-assigned gender. On the other hand, previous data demonstrated a normalization of absolute bone mineral density and/or *z*-scores during gender-affirming hormone treatment (Delemarre-van de Waal & Cohen-Kettenis, 2006; Mueller et al., 2005). However, studies focusing on the long-term effects of GnRH analogs on bone mineral density are scarce. Other concerns are related to possible adverse cardiovascular events associated with gender-affirming hormone therapy (Martinez et al., 2020; Ocampo-Serna et al., 2020). Some authors found an elevated risk of venous thromboembolism in trans* females under gender-affirming hormone therapy, compared to cis female controls without hormone treatment (Getahun et al., 2018). Confounding risk factors, such as substance use, infections, or smoking, are rarely included in these studies, some of which are known to be more prevalent in the trans* community (World Health Organization, 2015). Other areas where more evidence is needed include sexual dysfunction, pelvic pain, pelvic inflammatory disease, infertility, and neurocognition (Baxendale, 2024). While it is beyond this study’s scope to make statements about aspects related to somatic health, they should be considered carefully by clinicians and researchers.

### Limitations

A major limitation of this study is its naturalistic design, which involved non-randomized, retrospective group assignment and resulted in unequal group sizes. Specifically, the group of participants with no gender-affirming hormones or surgery was relatively small. We are also aware of the fact that, because of missing randomization, group affiliation might have been influenced by undetected determinants and thus biased. Also, birth-assigned females were overrepresented. These proportions, however, reflect the general demographic of those seeking gender identity healthcare. Three separate timepoints for each individual (before medical intervention, after gender-affirming hormones, and after gender-affirming surgery) would have enabled a more accurate assessment of the additional effect of surgery on body satisfaction. In addition, because of the small sample size, adolescents with pubertal blockade were included in the untreated group. Early puberty blockade may substantially influence body satisfaction, so in future studies, this group should be investigated separately. Furthermore, our data come from questionnaires and interviews. BIS is a unidimensional measure that does not account for emotion toward one’s embodiment and therefore does not capture all facets of body satisfaction. More qualitative research may possibly better capture the complexity of body satisfaction in transgender persons (McGuire et al., 2016). Another limitation of this study is that no data were collected about whether participants had reached their definitive treatment goals at timepoint T1, potentially influencing body satisfaction levels. The concepts of clinical effects/responses and statistical effects are both important (Pintea, 2010). Clinical effects refer to the meaningful real-world impact of treatment on a patient’s health or well-being, while statistical effects pertain to the significance of these effects as determined by statistical analysis, indicating whether they are likely due to the treatment rather than chance. While there is no established benchmark for clinical improvement on the BIS scale, the average improvements for the GAHT and GAHT + GAS groups in our study were 13% and 18%, respectively. (An improvement that might be of clinical importance for the patient but is not clear to be in the range of a defined response rate for this questionnaire.) The no-treatment group had lower mean body dissatisfaction and GD scores at baseline, which may have influenced why these participants were less likely to receive gender-affirming interventions. Also, the attendance of laser or speech therapy was not controlled for in the study, and this may have influenced participants’ body satisfaction. Another important topic for future studies is the role of psychiatric comorbidities in body satisfaction, as a negative body image is often associated with mental disorders (Scheffers et al., 2017). Furthermore, potential selection and response bias must be considered, given that our population attended a specialist gender identity service of a psychiatric clinic and that a portion of the original participants dropped out before the T1 assessment. These results may not necessarily generalize to unselected adolescents seeking gender identity services. It is also worth mentioning, when evaluating gender-affirming care, that some individuals do not continue on the path of gender transition and some voices advocate for more research on factors contributing to desistance (Butler & Hutchinson, 2020). Finally, this study only provides answers about the short-term effects of gender-affirming interventions, and future studies focusing on long-term satisfaction as well as the role of potential subsequent regret related to irreversible body changes are needed.

### Conclusions

This naturalistic study, despite its limitations, suggests that medical interventions, such as gender-affirming hormones and surgery, reduce body dissatisfaction in gender dysphoric youth. In addition, social transitioning and family functioning are important predictors of subsequent body satisfaction in gender dysphoric adolescents. Finally, body satisfaction was correlated with life satisfaction in this study. Further studies are needed to examine the specific effects, benefits, and risks of each gender-affirming treatment for gender dysphoric adolescents.

## Data Availability

Data are available upon a reasonable request.

## Code availability

Code is available upon a reasonable request.
